# Good news or bad news? The impact of information valence on high school students’ willingness to share misinformation and the effectiveness of a targeted accuracy prompt

**DOI:** 10.3389/fpsyg.2025.1664890

**Published:** 2025-10-28

**Authors:** Zhichao Wang, Hua Jin, Shanshan Li

**Affiliations:** ^1^School of Teacher Education, Shandong University of Aeronautics, Binzhou, China; ^2^Key Research Base of Humanities and Social Sciences of the Ministry of Education, Academy of Psychology and Behavior, Tianjin Normal University, Tianjin, China; ^3^Faculty of Psychology, Tianjin Normal University, Tianjin, China

**Keywords:** emotional valence, misinformation, adolescents, social media, information sharing, accuracy prompt

## Abstract

**Background:**

With the rapid proliferation of misinformation on social media, increasing attention has been paid to its psychological and behavioral mechanisms. Emotional valence—particularly the positive or negative tone of information—is often used in constructing misinformation, facilitating its wide dissemination. However, existing findings on how emotional valence influences misinformation sharing remain mixed, especially among adolescent populations. This study explores the impact of information valence on high school students’ willingness to share misinformation and evaluates the effectiveness of a targeted accuracy prompt.

**Methods:**

Two experiments were conducted. In Experiment 1, 53 high school students completed a news-sharing task involving both true and false headlines with varying emotional valence. Their willingness to share was measured. In Experiment 2, 40 students received a valence-targeted accuracy prompt designed to highlight common characteristics of misinformation. The effectiveness of the intervention in reducing misinformation sharing was then assessed.

**Results:**

Experiment 1 showed that participants were significantly more willing to share positive misinformation than negative misinformation, regardless of authenticity. Information valence had a significant effect on response bias. In Experiment 2, students who received the accuracy prompt intervention demonstrated significantly lower willingness to share misinformation compared to the control group, indicating the effectiveness of this brief and targeted approach.

**Conclusion:**

Information valence plays a critical role in shaping adolescents’ willingness to share misinformation, with positive content being more readily shared. A brief accuracy prompt intervention tailored to information characteristics and emotional valence can effectively reduce misinformation sharing among high school students. These findings provide theoretical and practical insights into combating misinformation in adolescent populations.

## 1 Introduction

In the digital era, the rapid spread of misinformation has emerged as a serious concern within online networks. Compared with factual information, misinformation tends to propagate more rapidly and widely, resembling the viral nature of a contagion (Vosoughi et al., 2018). Individuals are easily influenced by misinformation and often contribute to its diffusion by sharing it with others (Keselman et al., 2021). Lim and Perrault (2020) argue that the core issue lies more in the act of sharing than in the creation of misinformation, noting that its potential harm remains limited unless it garners public attention. The widespread dissemination of misinformation presents significant challenges to informed decision-making, particularly in critical domains such as public health (Apuke and Omar, 2021; Lewandowsky et al., 2012; Pennycook et al., 2020b; Vogel, 2017) and emergency management (Gupta et al., 2013; Keim and Noji, 2011; Spiro et al., 2012), thereby underscoring the urgency and necessity of research into the mechanisms underlying misinformation sharing.

A common feature of misinformation is the use of emotional language and appeals designed to provoke emotional reactions, which significantly contribute to its virality (Vosoughi et al., 2018; Chen et al., 2021; Igwebuike and Chimuanya, 2021; Kumar et al., 2021; Song et al., 2019). Brady et al. (2020) explored the psychological mechanisms behind the rapid spread of moral and emotional content, finding that such material tends to capture attention and correlates strongly with sharing behaviors on social media. Emotional characteristics—especially valence and arousal—play a pivotal role in influencing individuals’ willingness to share Berger and Milkman (2012), through a large-scale analysis of nearly 7,000 *New York Times* articles and subsequent experimental studies, concluded that content with positive emotional valence increases the likelihood of sharing. People are more inclined to disseminate content that is entertaining, helpful, or emotionally uplifting, driven by a preference to be perceived as spreaders of optimistic messages rather than sources of upsetting or anger-inducing content. Positive sharing can elevate others’ moods and yield social rewards.

However, this perspective is not universally accepted. Other studies suggest that audiences show a greater interest in negative content, including crises, conflicts, and disasters (Galil and Soffer, 2011; Soroka and McAdams, 2015). Salgado and Bobba (2019) found that negatively toned content on Facebook generates higher user engagement. Supporting this notion, Soroka et al. (2019) conducted an experimental study across 17 countries, revealing a consistent negativity bias: participants presented greater heart rate variability (HRV) in response to bad news compared to positive news. This finding implies that negative content is more attention-grabbing and that the preference for such content is cross-culturally robust. These conflicting findings indicate that the emotional valence of content—both positive and negative—merits deeper exploration in relation to misinformation sharing.

Among various interventions to combat misinformation targeted at adolescents, psychological inoculation (Schubatzky and Haagen-Schützenhöfer, 2023) and indicator-based approaches (Hartwig et al., 2024) are commonly employed, yet both face significant limitations. While effective, psychological inoculation interventions are often time-intensive and may fail to engage those with lower cognitive reflection—the very group most vulnerable to misinformation (Pennycook and Rand, 2021). Indicator-based interventions, on the other hand, require large-scale content monitoring and labeling, which is often impractical and may trigger implied truth effect through repeated exposure to flags (Pennycook et al., 2020a). These challenges are particularly acute for adolescents, who frequently exhibit underdeveloped critical thinking skills, overconfidence, and high exposure to online misinformation due to extensive social media use (Papapicco et al., 2022). Their heightened vulnerability calls for more feasible and scalable interventions. In this context, targeted accuracy prompts—which deliver concise, cues focused on specific features of misinformation—represent a promising alternative. Prompting accuracy before sharing can reduce the spread of misinformation, a conclusion supported by causal evidence from survey experiments and field studies on Twitter (Pennycook et al., 2020b, 2021). Although accuracy prompts have been recognized as an effective intervention, studies such as Gawronski et al. (2023) indicate that their effect sizes tend to be small, potentially due to a lack of specificity in targeting misinformation. Therefore, this study adopts a targeted accuracy prompt approach, focusing on precise cues related to characteristics that may influence adolescents’ sharing of misinformation. Such prompts are not only easy to implement and scale on social media platforms but may also prove more effective for adolescents than generic reminders, as they reduce cognitive load and directly guide attention toward unreliable content.

This study aims to address these gaps by investigating the effect of emotional valence on adolescents’ willingness to share misinformation and evaluating the effectiveness of accuracy prompt interventions. While considerable research has focused on adult behavior, adolescent sharing of misinformation remains underexplored. This is a critical oversight, especially given that, as of June 2024, China had 188 million underage internet users, accounting for 17.1% of its total online population (according to the 54th *China Statistical Report on internet Development*) (China Internet Network Information Center, 2024). Adolescents are at a unique stage of cognitive and emotional development. Their cognitive control abilities are still maturing (Casey et al., 2008), while their social–emotional neural systems —including the amygdala, ventral striatum, orbitofrontal cortex, and medial prefrontal cortex—are highly active (Steinberg, 2010), often leading to emotionally-driven and impaired decision-making (van Duijvenvoorde et al., 2010). Furthermore, adolescents frequently lack the digital literacy and social experience necessary to navigate the complex online information landscape. Given the continued prevalence of misinformation on social media (as noted by the China Internet Joint Rumor-Refutation Platform) (Cyberspace Administration of China, 2024), many adolescents remain ill-equipped to verify information authenticity or utilize fact-checking tools effectively (China Youth Internet Association, 2024).

Therefore, the present study investigates how the emotional valence of information influences high school students’ willingness to share misinformation. Utilizing the true-false news-sharing task paradigm developed by Pennycook et al. (2020b, 2021), we examine adolescent responses within a social media context to identify patterns in misinformation sharing. Building upon these insights, we develop and assess targeted accuracy prompt interventions based on information valence, aiming to reduce the willingness of adolescents to spread misinformation and to contribute practical solutions to the ongoing challenge of digital misinformation.

## 2 Experiment 1

This experiment aimed to investigate the impact of emotional valence on high school students’ willingness to share misinformation by comparing their willingness to share true versus false news under different valence conditions. A 2 (authenticity: true vs. false) × 2 (emotional valence: positive vs. negative) within-subjects design was employed. All participants were exposed to both positive and negative news items. After viewing each item, they were asked to rate their willingness to share it.

### 2.1 Methods

#### 2.1.1 Participants and design

The required sample size was estimated using G*Power 3.1.9.2 software. Based on a small-to-medium effect size (*f* = 0.20, with *α* = 0.05 and 1 − *β* = 0.80), the minimum required sample size was determined to be 36. To meet this requirement, 56 high school students (28 males and 28 females; age range: 16–18 years; *M* = 16.08, *SD* = 0.51) with normal or corrected-to-normal vision. The participants selected for the experiment were all self-reported active social media users who regularly share content online. Those who were inactive on social media or engaged in sharing less than once per week were excluded from the study. All participants provided informed consent, and the study was approved by the Ethics Committee of Shandong University of Aeronautics.

The experiment utilized a 2 × 2 within-subjects design. In addition to examining news-sharing intentions, signal detection theory (SDT) was applied to assess participants’ sensitivity to information authenticity. Following the approach of Batailler et al. (2022), we measured discrimination sensitivity (*d′*) and response bias (*c*) to provide nuanced insights into participants’ decision-making regarding information sharing. In our analysis, we opted to use the response bias metric *c*. Although both *d’* and *c* are derived from hit rates and false alarm rates, these two metrics are conceptually independent of each other (Macmillan and Creelman, 2004; Stanislav and Todorov, 1999).

The dependent variables primarily include the following: (1) Sharing Intention: participants rated their willingness to share each news item on a 6-point Likert scale (1 = extremely unlikely to share, 6 = extremely likely to share). (2) Discrimination sensitivity (*d’*): Calculated as *d′* = *z*(*H*) − z(FA), where *H* refers to the proportion of true headlines participants chose to share, and FA refers to the proportion of false headlines shared. (3) Response bias (*c*): Calculated as *c* = − $\frac{1}{2}$ ×[*z*(*H*) + *Z*(FA)]. Sharing intentions were binarized for SDT analysis (ratings 1–3 = “not share”; ratings 4–6 = “share”).

#### 2.1.2 Materials

True and false headlines were selected based on established methods (Pennycook et al., 2020b, 2021). True headlines were sourced from reputable media outlets such as Xinhua News and People’s Daily Online, while false headlines were verified as misinformation by platforms including the China Internet Joint Rumor-Refutation Platform, Xinhua News Agency App, and Tencent Jiaozhen.

**Figure 1:**
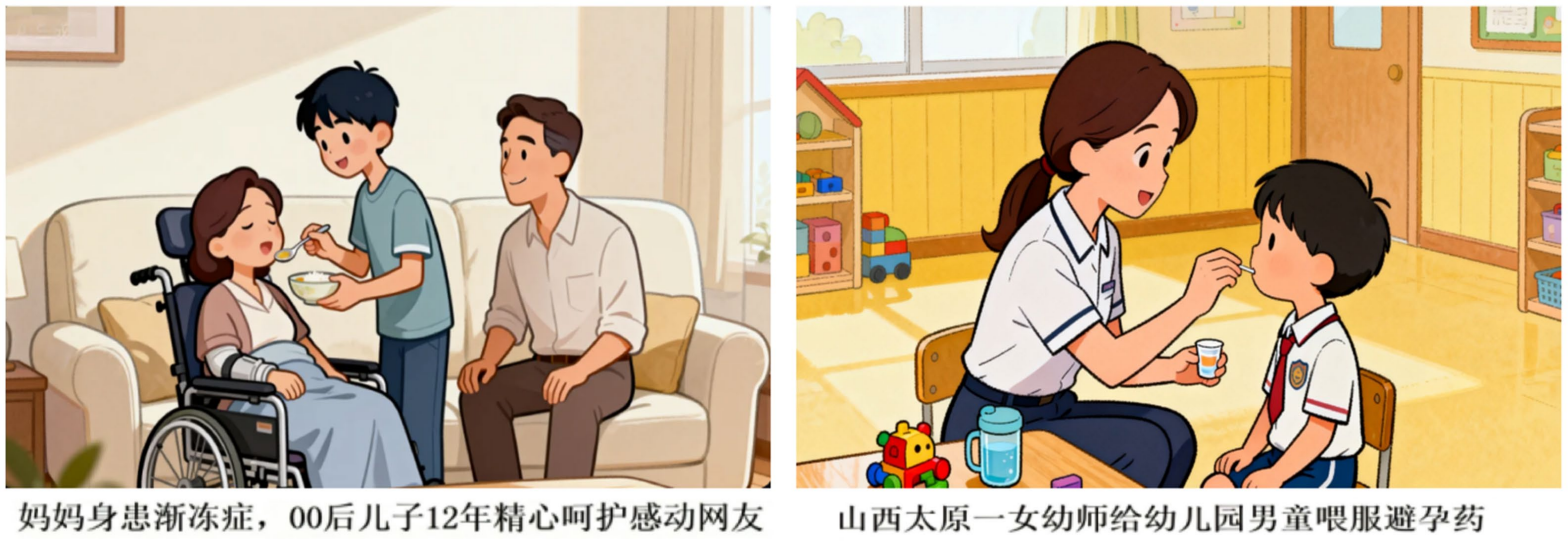
Sample headlines (the left panel presents a real positive news headline: *Chinese Gen Z Son’s 12-Year ALS Care for Mother Touches Millions*. And the right displays a false negative news headline: *Shanxi Kindergarten Teacher Allegedly Force-Feeds Birth Control Pills to Male Pupil*).

An initial pool of 30 true and 30 false headlines was evaluated by 40 high school students on valence (1 = very negative; 7 = very positive), arousal (1 = very calm; 7 = very excited), and familiarity (1 = very unfamiliar; 7 = very familiar). Based on these ratings, 40 headlines were selected for the formal experiment (20 true, 20 false), equally split between positive and negative valence. Each was presented in a “picture + headline” format mimicking real social media content (Sample headlines see Figure 1). The visual stimuli presented in the figures of this manuscript are AI-generated illustrations created for the purpose of publication. They serve as scientifically accurate representations of the original stimulus set used during data collection, which could not be published due to copyright restrictions. All data analyses, results, and conclusions are based solely on the participants’ responses to the original stimuli.

Independent sample *t*-test confirmed significant differences in valence for both true and false headlines. For true headlines, *t*(18) = 33.19, *p* < 0.001 (positive headline valence: *M* = 6.09, *SD* = 0.34; negative headline valence: *M* = 2.29, *SD* = 0.13); for false headlines, *t*(18) = 15.86, *p* < 0.001 (positive headline valence: *M* = 5.93, *SD* = 0.50; negative headline valence: *M* = 2.47, *SD* = 0.47). For both true and false headlines, there were no significant differences in arousal or familiarly ratings between positive and negative headlines for either authenticity type. In terms of headline arousal levels, for true headlines, *t*(18) = 0.54, *p* > 0.05 (positive headline: *M* = 4.86, *SD* = 0.59; negative headline: *M* = 5.01, *SD* = 0.70); for false headlines, *t*(18) = 0.07, *p* > 0.05 (positive headline: *M* = 4.81, *SD* = 0.93; negative headline: *M* = 4.84, *SD* = 0.45). In terms of headline familiarity levels, for true headlines, *t*(18) = 0.47, *p* > 0.05 (positive headline: *M* = 4.12, *SD* = 0.70; negative headline: *M* = 4.27, *SD* = 0.72); for false headlines, *t*(18) = 0.47, *p* > 0.05 (positive headline: *M* = 4.20, *SD* = 0.78; negative headline: *M* = 4.04, *SD* = 0.79).

#### 2.1.3 Procedure

Participants were informed they would be evaluating real social media news screenshots. The experimental was administered using E-prime 2.0 program. Each trial began with a fixation cross “+” displayed for 1,000 ms, followed by a news screenshot. Participants then rated their willingness to share it using the 6-point scale. The next item was presented automatically upon response. The session lasted approximately 10–15 min. Responses were automatically recorded.

Three participants were excluded due to inattentive responses (mean reaction time <500 ms), resulting in a final sample of 53 participants (25 males, 28 females).

### 2.2 Results

A 2 (authenticity: true vs. false) × 2 (valence: positive vs. negative) repeated-measures ANOVA was conducted. The main effect of authenticity was not significant, *F*(1, 52) = 0.35, *p* > 0.05, *η_p_*^2^ = 0.01. However, the main effect of valence was significant, *F*(1, 52) = 17.44, *p* < 0.001, *η_p_*^2^ = 0.25, indicating that participants were more willing to share positive headlines (*M* = 3.66, *SD* = 1.04) than negative ones (*M* = 3.00, *SD* = 0.98).

**Figure 2:**
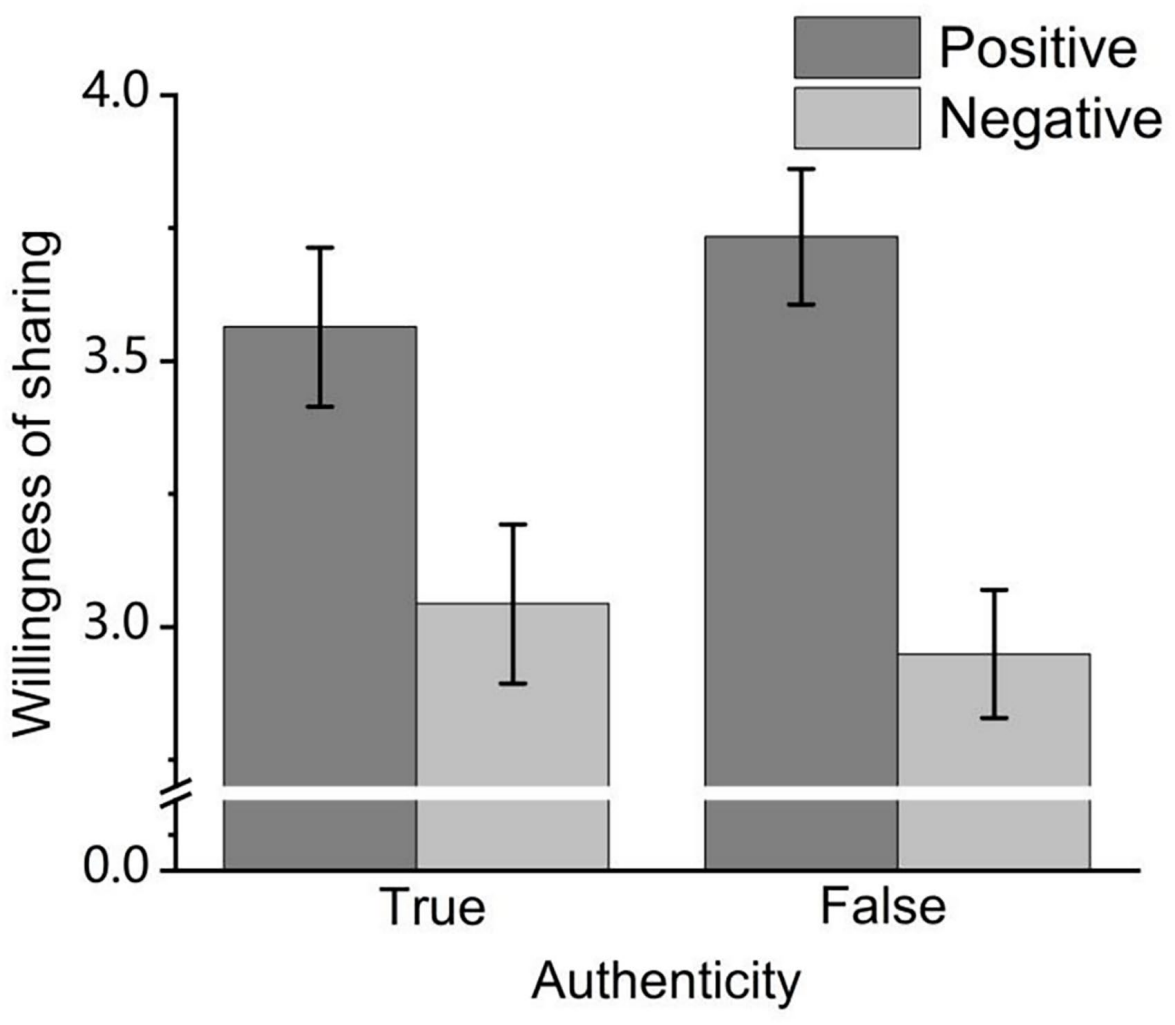
Mean willingness to share headlines across different valences in Experiment 1.

The interaction between authenticity and valence was marginally significant, *F*(1, 52) = 3.89, *p* = 0.054, *η_p_*^2^ = 0.07. Simple effect analyses revealed significant differences in sharing intention between positive and negative headlines for both true, *F*(1, 52) = 7.03, *p* < 0.05, *η_p_*^2^ = 0.12 and false [*F*(1, 52) = 31.91, *p* < 0.001, *η_p_*^2^ = 0.38] conditions, with a stronger effect observed for false headlines (See Figure 2).

Subsequently, discrimination sensitivity (*d’*) and response bias (*c*) were calculated, followed by regression analysis to examine the effect of emotional valence on these two indicators. The results showed that emotional valence did not significantly predict discrimination sensitivity, *β* = −0.09, *F*(1, 104) = 0.76, *p* > 0.05. However, emotional valence had a significant effect on response bias, *β* = −0.33, *F*(1, 104) = 12.26, *p* < 0.01. Specifically, as the emotional valence of information became more positive, the response bias value significantly decreased. A positive value of *c* reflects a conservative bias (i.e., a tendency not to share, regardless of authenticity), while a negative value of c reflects a liberal bias (i.e., a tendency to share, regardless of authenticity). This suggests that participants were more inclined to share information when it was positively valenced, irrespective of whether it was true or false.

To validate the robustness of the binarized classification approach, we performed ROC curve analysis. The results showed that the AUC for the positive condition was 0.627 (95% CI: 0.521–0.733), while for the negative condition it was 0.663 (95% CI, 0.560–0.766). The difference in AUC between the two conditions was not significant (0.036, *p* = 0.632). These findings support the hypothesis that valence primarily affects response bias rather than discriminative sensitivity. Specifically, the positive condition exhibited a liberal response bias (*c* = −0.357), whereas the negative condition showed a conservative response bias (*c* = 0.419), with a significant difference between them (0.776). Threshold sensitivity analysis further confirmed the rationality of using the 3.5 cutoff, indicating that the binarized classification method possesses good robustness.

### 2.3 Discussion of experiment 1

The findings of Experiment 1 revealed that information authenticity did not significantly affect sharing intentions, which is consistent with previous research indicating that individuals often struggle to differentiate between true and false information when deciding what to share online. More importantly, emotional valence significantly influenced high school students’ willingness to share misinformation, with participants showing a significantly higher willingness to share positive headlines compared to negative ones. This aligns with earlier studies suggesting that emotionally positive content is more likely to be shared (Berger and Milkman, 2012), and the current results extend this pattern to the adolescent population.

Although the interaction between authenticity and valence was only marginally significant, further simple effect analyses revealed that the difference in willingness to share positive vs. negative headlines was significant for both true and false information. Notably, this effect was stronger for false headlines, suggesting that positively-valenced misinformation may be especially compelling and likely to be shared by high school students. This pattern may be explained by the entertaining or altruistic qualities of some of the positively framed misinformation used in the study—for example, stories about miraculous rescues, community donations, or heartwarming technological advancements—which may make them more emotionally engaging or socially rewarding to share.

Ceylan et al. (2023) posit that misinformation sharing is fundamentally habitual—a perspective offering explanatory power for the current findings. Adolescents’ preference for sharing positive-content may reflect not merely valence-based evaluation, but more fundamentally, affectively cued habitual automation. This aligns with the habit-goal interface theory (Wood and Rünger, 2016): positive valence serves as a contextual trigger activating preexisting sharing scripts, with the “single-tap sharing” affordance of social media platforms reinforcing habit loops. Receiving positive feedback (e.g., like notifications) on social media activates reward-processing regions including the striatum and ventral tegmental area (Sherman et al., 2018). Crucially, adolescents’ heightened sensitivity to peer evaluation motivates impression management through sharing positive-content, this may form a situation-response connection, causing sharing behaviors to break away from prudent evaluation.

Additionally, the results from the signal detection theory (SDT) analysis provide further insight: while emotional valence did not significantly impact participants’ ability to distinguish true from false information (i.e., discrimination sensitivity, *d′*), it did have a significant effect on their response bias. Specifically, participants demonstrated a stronger bias toward sharing positively valenced information, regardless of its truthfulness. This suggests that even when individuals are capable of identifying false information, they may still choose to share it if it carries a positive emotional tone—potentially prioritizing emotional resonance or social value over accuracy. This tendency contributes to the amplification and spread of misinformation on social media platforms.

## 3 Experiment 2

Experiment 1 demonstrated that emotional valence significantly influences high school students’ willingness to share misinformation, with a preference for sharing positively-valenced headlines. These results suggest that adolescents may overlook the authenticity of information when deciding to share it. Experiment 2 aimed to test the effectiveness of an accuracy prompt intervention, in which participants were encouraged to consider the accuracy of emotionally positive headlines before making sharing decisions.

### 3.1 Methods

#### 3.1.1 Participants and design

The required sample size was estimated using G*Power 3.1.9.2 software. Assuming a small-to-medium effect size (*f* = 0.20), with *α* = 0.05 and (1 − *β*) = 0.80, the analysis yield a minimum required sample size of 36 participants. To meet this requirement and allow for possible exclusions, 40 high school students (18 males, 22 females; *M* = 15.53 years, *SD* = 0.68, age range: 15–17) were recruited, none of whom had participated in Experiment 1. All participants had normal or corrected-to-normal vision. The participants selected for the experiment were all self-reported active social media users who regularly share content online. Those who were inactive on social media or engaged in sharing less than once per week were excluded from the study. Informed consent was obtained prior to the experiment, and ethical approval was granted by the Ethics Committee of Shandong University of Aeronautics.

The experiment used a 2 (Authenticity: true vs. false) × 2 (Intervention: pre- vs. post-intervention) within-subjects design. Each participant read 20 headlines divided into two phases: 10 headlines were presented without intervention, and the remaining 10 with the accuracy prompt intervention. The dependent variable was willingness to share each headline on social media, rated on a 6-point Likert scale (1 = extremely unlikely, 6 = extremely likely).

#### 3.1.2 Materials

**Figure 3:**
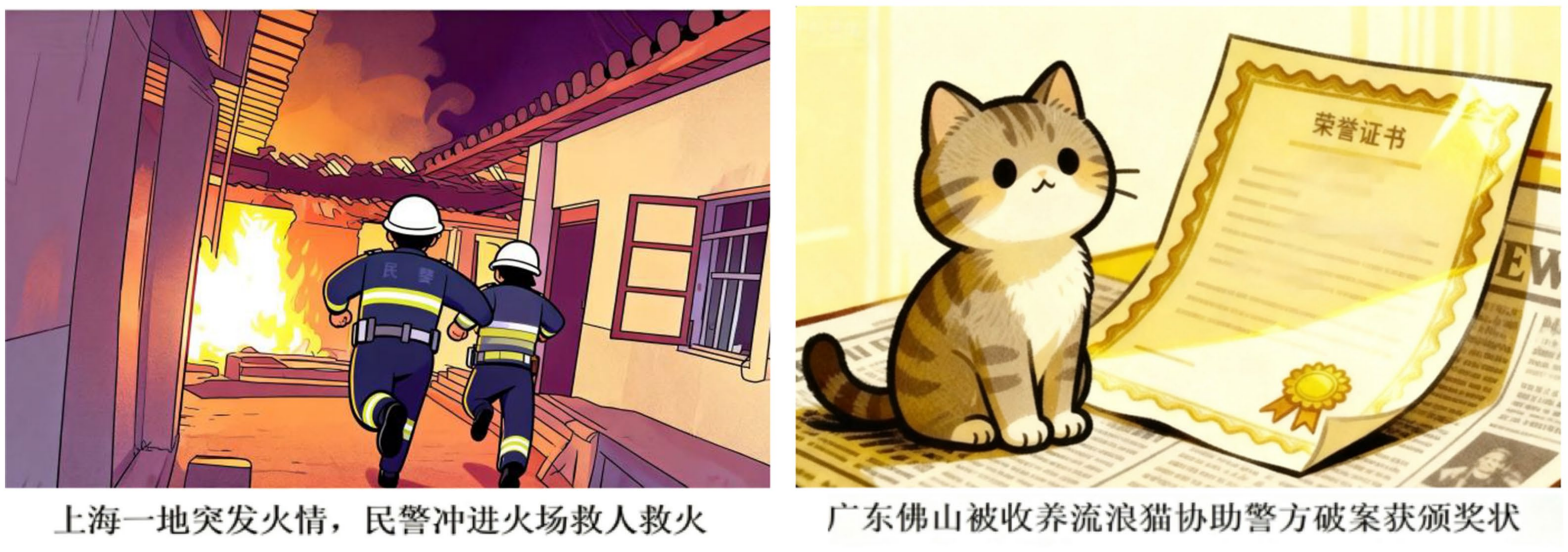
Sample headlines (the left panel presents a real positive news headline: *Police Dash into Burning Building to Rescue Residents, Fight Blaze in Shanghai*. And the right displays a false positive news headline: *“Claw-enforcement”: Adopted Stray Cat in Foshan Helps Crack Case, Gets Award*).

The headlines used in this study were selected from Experiment 1. Given that participants exhibited a significantly higher willingness to share positive headlines, the intervention focused solely on positive content to avoid floor effects associated with negative headlines. A total of 20 positive headlines (10 true, 10 false) were included, each formatted as a screenshot combining an image and a headline, mimicking typical social media posts (Sample headlines see Figure 3).

#### 3.1.3 Procedure

Participants were informed that they would be viewing screenshots of information from social media. The experiment was administered using E-Prime 2.0 software and conducted in two phases.

In Phase 1 (pre-intervention), a fixation cross (“+”) appeared for 1,000 ms, followed by presentation of the first 10 headlines. After each headline, participants rated their willingness to share using the 6-point scale. Once a response was made, the next headline appeared automatically.

In Phase 2 (post-intervention), participants first read the following instruction:” *Some information on social media is true, while some is false. False information is sometimes especially positive and appealing. Please carefully scrutinize such content before deciding to share*.” Following this, the remaining 10 headlines were presented, and participants again rated their willingness to share each item.

To control for order effects, each headline appeared with equal frequency across the two phases, and their presentation order was fully counterbalanced across all participants. Specifically, each headline was presented in the pre-intervention phase for half of the participants and in the post-intervention phase for the other half. The entire task took approximately 5–10 min, and responses were recorded automatically.

### 3.2 Results

**Figure 4:**
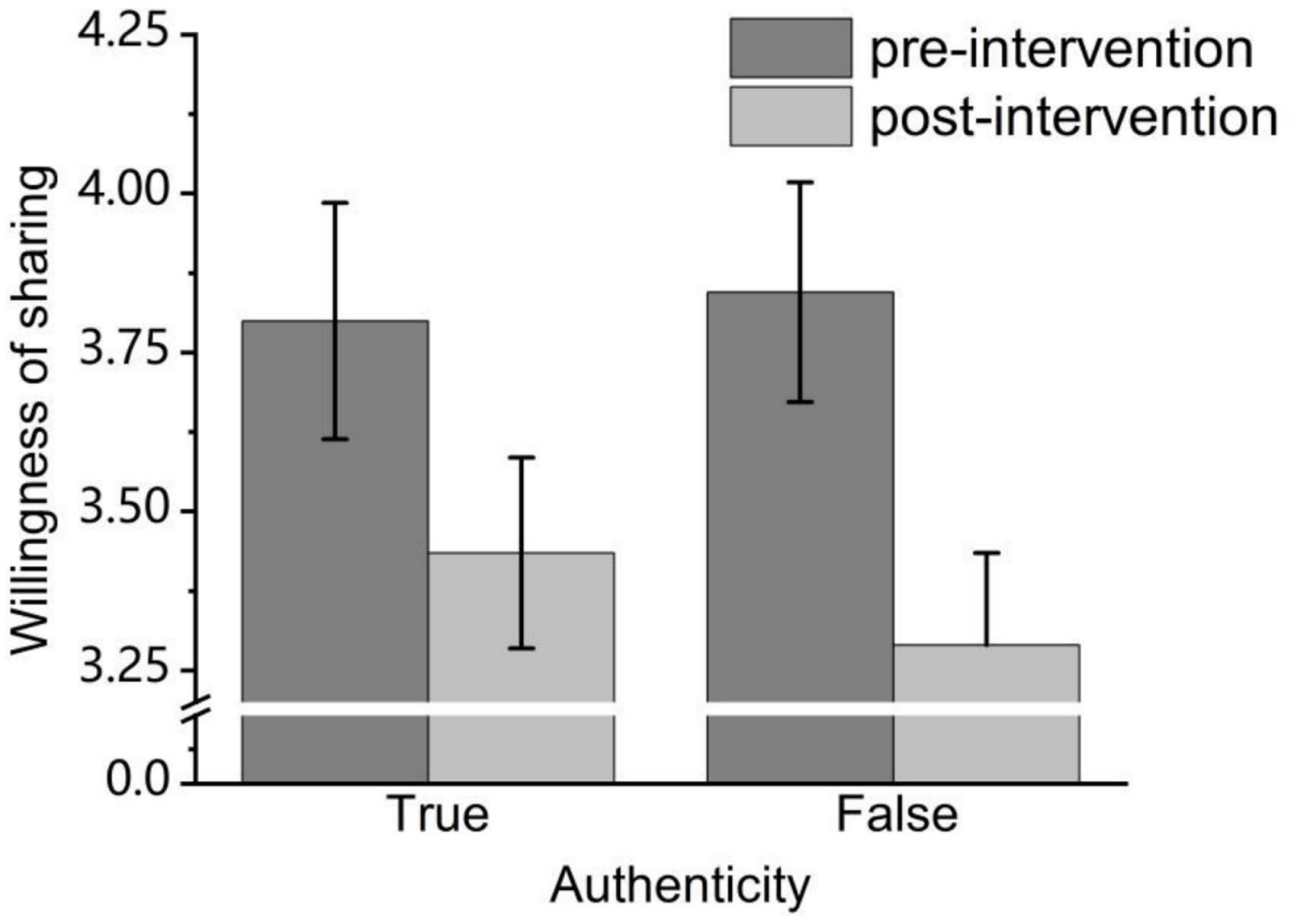
Mean willingness to share headlines across different conditions in Experiment 2.

A 2 (Authenticity: true vs. false) × 2 (Intervention: none vs. accuracy prompt) repeated-measures ANOVA was conducted. The results showed that the main effect of authenticity was not significant, *F*(1, 39) = 0.23, *p* > 0.05, *η_p_*^2^ = 0.006; the main effect of the intervention was significant, *F*(1, 39) = 12.56, *p* < 0.05, *η_p_*^2^ = 0.24, 95% CI [0.20, 0.72], which constitutes a large effect size according to Cohen’s (1988) standards. With the willingness to share headlines being significantly lower in the accuracy prompt condition than in the control condition. Specifically, the willingness to share false headlines decreased from 3.85 under the control conditions to 3.29 (average), and the willingness to share real information decreased from 3.80 under the control conditions to 3.44. The interaction effect between authenticity and intervention was not significant (See Figure 4).

Subsequently, discrimination sensitivity (*d’*) and response bias (*c*) were calculated, followed by regression analysis to examine the effect of accuracy prompt intervention these two indicators. The results showed that accuracy prompt intervention did not significantly predict discrimination sensitivity, *β* = −0.01, *F*(1, 78) = 0.02, *p* > 0.05. However, accuracy prompt intervention had a significant effect on response bias, *β* = −0.34, *F*(1, 78) = 10.49, *p* < 0.01. This indicates that the accuracy prompt intervention did not improve adolescents’ ability to distinguish between true and false information, and generally reduced their intention to share positive content, regardless of whether it was true or false.

### 3.3 Discussion of experiment 2

The findings from Experiment 2 revealed that the accuracy prompt intervention significantly reduced participants’ willingness to share information, with a large effect size (*η_p_*^2^ = 0.24). This suggests that interventions which prompt individuals to reflect on information accuracy—especially in emotionally positive content—can effectively curb the spread of misinformation.

While the main effect of authenticity was not significant, this result may indicate a spillover effect of the intervention: participants became more cautious overall, reducing their willingness to share both false and true headlines. Although not statistically significant, the interaction trend suggests that the reduction in sharing was greater for false headlines, implying the intervention may be more effective in targeting misinformation. The results from SDT indicate that the primary mechanism of this intervention may not involve enhancing cognitive discriminability, but rather inducing a more cautious response bias in sharing behavior. This shift in response bias may hold particular value for adolescent populations, whose cognitive reflection abilities are still developing. Compared to interventions requiring complex cognitive processing and factual knowledge—such as inoculation or fact-checking—providing simple, specific sharing behavior prompts (e.g., targeting features like “positiveness”) better aligns with adolescents’ cognitive characteristics and imposes a lower cognitive load. Consequently, this approach may offer greater feasibility and scalability in real-world applications.

Besides, given that misinformation often poses a greater threat than the suppression of truthful content, this minor decrease in sharing true information might be a reasonable trade-off. In real-world social media contexts, such accuracy prompt based interventions could offer substantial societal benefits by effectively reducing the dissemination of misinformation among adolescents and potentially broader populations.

## 4 General discussion

This study conducted two experiments to examine the impact of emotional valence on high school students’ willingness to share misinformation, as well as the effectiveness of accuracy prompt interventions targeting emotional valence in reducing such willingness. Across both experimental studies, we implemented the information-sharing task while deliberately excluding requirements for truthfulness judgments from participants. Empirical research has shown that judging the truthfulness of information itself is an intervention, which can affect subsequent performance in information-sharing tasks (Pennycook et al., 2020b, 2021). Given that the truthfulness judgment task may not fully capture the relevant motivations driving information-sharing decisions in real social contexts, we only included the information-sharing task in our experimental design and did not require participants to judge the truthfulness of the information. The core purpose of this design is to maximally replicate individuals’ decision-making patterns in daily information interactions, reduce research biases caused by the disconnect between experimental task settings and real-world behaviors, thereby effectively enhancing the ecological validity of research conclusions in real social contexts, and ensuring that the research results can be more reliably generalized to the explanation and prediction of information dissemination behaviors in natural settings.

Results from Experiment 1 demonstrated that emotional valence significantly influences students’ sharing intentions, with a notably higher tendency to share positive information over negative information. This findings is consistent with previous research on emotional valence and sharing behavior (Berger and Milkman, 2012), and confirms that this effect is also present in the adolescent population. Experiment 2 revealed that accuracy prompt interventions targeting emotional valence significantly reduced students’ willingness to share misinformation. Sharing intentions under the accuracy prompt condition were markedly lower than those in the control condition, and the intervention showed a large effect size. Collectively, these findings contribute to the experimental literature on emotional valence and misinformation sharing, while also enriching research on misinformation interventions among adolescents.

Specifically, emotional valence influenced response tendencies: students were more likely to share positive content, regardless of its authenticity. Signal Detection Theory (SDT) analysis revealed that while emotional valence had no significant effect on discrimination sensitivity (*d’*), it significantly affected response bias (*c*), indicating a general tendency to favor sharing more emotionally positive content. This may be due to two key factors:

First, previous research has shown that positive emotions increase credulity, whereas negative emotions can enhance skepticism (Forgas, 2019). This is explained by the way emotions influence cognitive processing: positive emotions promote heuristic, fluency-based thinking, while negative emotions elicit more deliberate, data-driven processing strategies (Forgas and Eich, 2012; Fredrickson, 2001). Positive headlines may thus increase perceived credibility and facilitate intuitive processing, leading adolescents to share them more readily.

Second, social media sharing involves value-based decision-making. Research indicates that people weigh both content-related factors (Vosoughi et al., 2018; Cappella et al., 2015) and social influences, such as perceived norms and peer approval (Ihm and Kim, 2018; Scholz et al., 2020), when deciding to share information. According to Social exchange theory (SET), users seek intangible social rewards—like reputation—through online sharing (Radecki and Spiegel, 2020; Wu et al., 2006). Adolescents are particularly sensitive to peer evaluation (Oh and Syn, 2015; Watson and Friend, 1969; Jackson et al., 2002), and often fear negative social judgment. Consequently, sharing positive information can enhance social perception and mood elevation, motivating adolescents to prioritize social rewards over informational accuracy.

Consequently, sharing positive information can lead to more favorable social evaluations, which in turn encourages adolescents to share more positive content while avoiding negative information. Moreover, disseminating positive content may uplift others’ moods or yield social rewards (Berger and Milkman, 2012). This value-driven motivation may outweigh the desire for accuracy, prompting individuals to share positively biased misinformation.

The interaction between authenticity and information valence was marginally significant. A simple effects analysis revealed that participants’ willingness to share both positive and negative information varied significantly for both true and false headlines, with stronger significance observed for false headlines. This suggests that perceived authenticity influences the sharing of misinformation among high school students. One possible reason for the significantly higher sharing of positive false information compared to negative false information is the entertaining or altruistic nature of some of the positive content used in the study. Prior research has indicated that when information is entertaining (Altay et al., 2022) or perceived as helpful to others, individuals are more likely to share it without verifying its accuracy, believing it will not harm their reputation. This increases the likelihood of such content being shared on social media (Acerbi, 2019; Sampat and Raj, 2022). As a result, even if individuals can discern between true and false headlines, they may prioritize emotional or social value over factual accuracy when sharing positive information—contributing to the spread of misinformation.

Experiment 2 employed an intervention method involving targeted accuracy prompt. Unlike the accuracy prompt used by Pennycook et al. (2020b, 2021), the accuracy prompt in this study explicitly highlighted that positive content is more likely to be misinformation, making the intervention more targeted than previous approaches. This study employed targeted accuracy prompt, which significantly reduced adolescents’ intention to share misinformation. Moreover, the relatively large effect size demonstrates that targeted accuracy reminders yield better intervention outcomes. Although the intervention did show some spillover effects, slightly reducing willingness to share true information, the reduction was smaller than that for misinformation. This intervention can be regarded as a ‘harm minimization’ strategy—it indirectly reduces opportunities for misinformation to spread by dampening the overall impulse to share, particularly for content with the greatest potential to go viral. Given the greater harm posed by misinformation, this trade-off is acceptable. With its relatively large effect size, the accuracy prompt approach—if applied to real-world social media environments—could yield substantial societal benefits by curbing the dissemination of false information.

The key strengths of this characteristic-based accuracy prompt intervention lie in its simplicity, clarity, and scalability. It only requires brief reminders about accuracy and prompts users to recognize specific features indicative of misinformation. This makes it easily adaptable to real-world social media contexts and suitable for broad implementation across platforms. Furthermore, while the intervention educates users on which content traits may signal misinformation, it still preserves individual autonomy by allowing users to make their own decisions about engagement and sharing. Therefore, characteristic-based accuracy prompt represents a highly promising strategy for mitigating the spread of misinformation online.

While the promising nature of such interventions often warrants investigation into their long-term effects, this study focused on immediate outcomes rather than incorporating a follow-up design—a common approach in many existing intervention studies (Miri et al., 2024). This approach was driven by our primary considerations of enhancing ecological validity and controlling response bias. Since the study participants were adolescents—a group particularly susceptible to evaluation apprehension and social desirability concerns (Jackson et al., 2002; Ollendick and Hirshfeld-Becker, 2002)—we implemented a rigorous anonymous protocol to ensure response authenticity, which, however, made subsequent tracking unfeasible. Furthermore, conducting longitudinal follow-ups outside the classroom setting with high school students posed practical challenges. This study follows the research approach of pioneering work in the field (Pennycook et al., 2020b), which holds that demonstrating a significant immediate effect is a necessary foundation for exploring long-term utility. Thus, the present study provides a valid and rigorous evaluation of the preliminary effectiveness of the intervention. Of course, we acknowledge that the absence of follow-up data somewhat limits inferences regarding the persistence of the effects. Future research could seek to overcome these obstacles by developing longitudinal designs in collaboration with educational institutions to further examine the long-term sustainability of the intervention effects.

In summary, this study used experimental methods to investigate how emotional valence influences adolescents’ willingness to share misinformation, and tested the effectiveness of a characteristic-based accuracy prompt intervention. These findings extend current research on misinformation sharing, enrich our understanding of influencing factors, and provide a foundation for developing more effective intervention strategies.

However, several limitations remain. First, this study used social media headlines and measured sharing intention rather than actual behavior. While previous research suggests that sharing intentions strongly predict actual sharing behavior—even differing little between those who click headlines or not, and that headlines suffice for accuracy judgments—these findings are based on adults (Mosleh et al., 2020; Molina et al., 2021), and their applicability to adolescents remains unclear. Although methodologically similar studies have shown generalizability to real-world behavior (Pennycook et al., 2021; Sampat and Raj, 2022), adolescent-specific factors like social desirability bias may limit such extrapolation. Future research should examine whether these results extend to other formats (e.g., full posts) in ecologically valid social media settings.

Second, while characteristic-based accuracy prompt reduced adolescents’ misinformation-sharing willingness, its spillover effect on true information requires mitigation strategies. Moreover, given adolescents’ underdeveloped information-discernment capacities and the spillover effects of interventions on genuine information-sharing, comprehensive strategies—such as fact-checking, rumor debunking, peer-evaluation sensitivity combined with social norm interventions, and media literacy cultivation—can collectively reduce misinformation sharing while curbing its dissemination.

Third, limited sample size constrains generalizability. Although G*Power-calculated sampling and homogeneous selection (active social media users with regular sharing) bolstered internal validity, restricted sample representativeness may attenuate statistical power for subtle effects, necessitating further verification of external validity.

Finally, the repeated-measures ANOVA employed in this study provided a valid foundation for testing the main effects, though it should be noted that this analytical approach may have certain limitations in capturing the hierarchical nature of the data. In experimental designs involving both participant- and item-related variability, incorporating mixed-effects models (such as linear mixed models or cumulative link models with random effects for participants and items) could offer additional insights for statistical inference. Such models have the potential to better accommodate multi-level data structures, and future studies may consider adopting these approaches to further advance related investigations.

## 5 Conclusion

This study provides preliminary evidence for the influence of emotional valence on adolescents’ willingness to share misinformation. High school students were significantly more inclined to share positive content than negative content, and emotional valence predicted a more liberal response bias, prompting users to share positively framed information regardless of authenticity. By introducing an accuracy prompt intervention that highlights emotional valence as a potential signal of misinformation, this study demonstrated a marked reduction in students’ sharing intentions. The large effect size suggests this method is both effective and scalable. As such, accuracy prompt strategies targeting content characteristics present a promising avenue for mitigating the spread of misinformation among adolescents in social media contexts.

## Data Availability

The raw data supporting the conclusions of this article will be made available by the authors, without undue reservation.
